# Incidence of human rabies virus exposure in northwestern Amhara, Ethiopia

**DOI:** 10.1186/s12879-018-3500-3

**Published:** 2018-11-26

**Authors:** Endalew Yizengaw, Tamyalew Getahun, Wondemagegn Mulu, Mulat Ashagrie, Ibrahim Abdela, Mekuanint Geta

**Affiliations:** 1 Department of Microbiology, Immunology and Parasitology, College of Medicine and Health Sciences, Bahir Dar University, Bahir Dar, Ethiopia; 2 Addis Alem Hospital, Bahir Dar, Ethiopia; 3 Department of Medical Microbiology, School of Biomedical and Laboratory Sciences, College of Medicine and Health Sciences, University of Gondar, Gondar, Ethiopia

**Keywords:** Rabies, Exposure, Human, Incidence, North-West Ethiopia

## Abstract

**Background:**

Clinical Rabies is a widely distributed almost 100% fatal viral zoonotic disease. Most human rabies cases occur in developing countries, especially in Asia and Africa. It can be prevented by immunization, post exposure prophylaxis. Ethiopia is assumed to be among African countries with high incidence of human rabies exposure cases. However, there is limited documented data on the incidence of human rabies exposure in the study area in particular and in Ethiopia in general. Thus, this study aimed to determine the Incidence of Human Rabies Virus exposure in Northwestern Amhara, Ethiopia.

**Methods:**

Retrospective cross-sectional study was conducted during August and September, 2017. The study was conducted at Addis Alem Hospital, a district level hospital in Bahir Dar. Data on human rabies exposure cases registered from September 1, 2015 to August 31, 2017 were collected from anti-rabies post exposure prophylaxis registration log book. Data was collected using a structured data collection questionnaire developed for this purpose. Descriptive statistics was used to describe relevant variables.

**The results:**

A total of 924 human rabies exposure cases was recorded. The overall human rabies incidence per 100, 000 population was 6.5 in 2015 and 7.5 in 2016. Males (55.2%, 510/924) and children of age less than fifteen (46.3%, 428/924) were most affected groups. The majority of human rabies exposure cases (71.9%, 664/924) were from rural settings. Dogs were the principal sources of exposure (96.3%) followed by cat (1.5%) and donkey (1.3%). High incidence rate of rabies exposure was reported during spring (360, 39%) and summer (244, 26.4%) seasons. There was significant difference between rural and urban exposure cases (*p* = 0.001) in respect to the time of arrival to the hospital.

**Conclusion:**

Taking these into account, a significant proportion of the population was exposed to rabies virus during the study years. There was high human rabies exposure rate in children and in the rural community.

This shows the need for organizing preventive and control strategies and to build community awareness.

## Background

Rabies, fatal but neglected disease, constitutes a major public health problem worldwide [1, 2]. The burden is so high in developing countries where access of preventive treatment is limited [3] with an annual mortality rate of over 60,000 of which Asia and Africa accounts 56 and 44% cases, respectively [2, 4, 5]. With this, rabies imposes an immense cost and hinders economic development with an estimated loss of 1.7 million daily adjusted life years and a global cost of 584 million US dollars [1, 6].

Rabies is a zoonotic disease affecting a wide range of wild and domestic animals, including livestocks [3, 4, 7]. Domestic dogs are the main sources of exposure and primary transmitter of human rabies, especially in African and Asian where there is no or inadequate dog rabies control strategies [7–10].

Rabies is almost always fatal acute viral disease of the central nervous system resulted from infection of neurotropic *Rabies virus* (RABV) [3, 11]. The *Rabies virus* is a bullet shaped single-stranded RNA virus that multiplies in the salivary glands of an infected host. Of different RABV species known to cause the disease, Rhabdoviridae family**,** genus Lyssavirus is responsible for the majority of cases [12, 13]. It is mostly transmitted by the saliva of infected animals, usually by biting and disease severity is determined by different factors like site of biting, extent of exposure, and species of the virus. The incubation period, normally ranges from 1 to 3 months, but can range from days to years. Prompt wound care and administration of post-exposure prophylaxis are highly effective in preventing clinical rabies [14].

Ethiopia is among high burden African countries in regard to human rabies virus exposure since ancient times [5, 14–18]. The Ethiopian Health and Nutrition Research Institute, the current Ethiopian Public Health Institute, indicated that human rabies has been reported in Ethiopia in 1903 for the first time. A national surveillance data conducted from 2007 to 2012 showed that 15,178 exposure cases (3.4/100,000), 272 fatal cases with more than 88% of the exposure cases were due to dog bites [19]. Most of the exposed cases (59.2%) were males. The majority of the exposure cases (98.9%) were from Addis Ababa, Oromia, Amhara, SNNPR, and Tigray regions. Tigray (11.4/100,000), Oromia (3.5/100,000), Benshangul Gumz (3.3/100,000), Amhara (1.5/100,000), SNNPR (1.2/100,000), and Addis Ababa (0.8/100,000 regions contributed the highest incidence of cases. Despite this information, there are no reliable data available on the annual number of people exposed to rabies. Thus, this study aimed to determine the incidence of human rabies exposure at Northwestern Amhara, North-West Ethiopia. Documentation of the epidemiological situation of rabies is required at the country level in general and in the study area in particular to show the impacts of the disease, evaluate control and prevention strategies and timely identify outbreaks and to plan future intervention and ultimate elimination strategies. This study also contributes beyond the country since it is an input to the current knowledge of the burden of rabies.

## Methods

### Study design and area

A Retrospective data review was done during August and September, 2017 on human rabies exposure cases registered from September 1, 2015 to August 31, 2017 at Addis Alem Hospital, a district level hospital, in Bahir Dar. Bahir Dar is the capital city of Amhara National Regional State (ANRS), Ethiopia. It is 570 km away from Addis Ababa in the North-west direction and situated in the south of Lake Tana, the biggest lake in the country. The hospital provides service for cases come from Bahir Dar City and other three zones (West Gojjam, Awi and South Gondar). The altitude ranges from 1799 m to 5902 ft. above sea level and the coordinates are 11°35.6184′ N latitude and 37°23.4462′ E longitude.

### Study population and data collection

Human rabies exposure cases, usually after biting by suspected rabid animals, including human beings, came or referred to Addis Alem Hospital. Exposure cases came to this hospital from four zones of the region to get post-exposure prophylaxis. We retrieved the records of 924 such exposure cases who were recorded for post exposure prophylaxis in the hospital between September 1, 2015 and August 31, 2017. The total number of human population at risk of human rabies exposure, for each respective year and respective zones included in this study, was estimated by projecting the results of the National Population and Housing Census of Ethiopia conducted in 2007. Two years data from registration log book were reviewed for demographic data; type of animal bite; date of arrival in the institution after exposure for rabies, season of exposure, residence of cases and health status of the animal involved in biting. More data on the different factors associated with human rabies was difficult because limited variables were used in the registration log book.

### Diagnosis of suspected dogs and human rabies exposure

Confirmatory laboratory tests to diagnose rabies are not available not only in the study hospital, but also around the study area. Rabies exposure was diagnosed clinically. For those cases who came to the hospital within three days of exposure, the dog involved in biting was quarantined and followed for ten days. The dog was followed for any changes in behavior and signs of rabid animal [2]. The dog was considered as rabid if it showed aggressive behavior; hydrophobia and/or more signs of a rabid animal. Strong history on the condition and the vaccination status of the dog was taken for those cases who came to the hospital after three days of exposure. Dogs involved in unprovoked biting, with signs of a rabid animal and/or unvaccinated were considered as rabid. All cases bitten by the suspected rabid dog as stated above were considered as rabies exposure cases. A suspected case is a case that is compatible with a clinical case definition and it is a probable case if it has a reliable history of contact with a suspected rabid animal [18]. The status for most of the exposure cases in this study was diagnosed by the later diagnosis method as most of them came to the hospital late and the dogs involved in biting were killed before quarantined and/or were stray dogs.

### Administration of Post exposure prophylaxis

Inactivated rabies virus (purified chick embryo cell) based post exposure prophylaxis (Rabipur®, NOVARTIS) is administered following procedures as per the world health organization. All bitten cases who came to the hospital were categorized as type I, type II and type III according to the European Centre for Disease Prevention and Control recommendation. All type II and type III cases with no sign of active disease received the post-exposure prophylaxis and there was no additional immunoglobulin for type III cases [20]. Type I exposure cases received the post-exposure prophylaxis only when they have reliable history. Exposure cases with minor scratches or abrasions without bleeding or licks on broken skin and nibbling of uncovered skin were classified as category II. Category III indicates cases with single or multiple transdermal bites, scratches, or contamination of mucous membrane with saliva.

### Data analysis

Data were entered and analyzed using SPSS Statistical software Package (IBM Corp. Released 2011 IBM SPSS statistics for windows, version 20. Armonk, NY: IBM Corp) Descriptive analysis was done to characterize and summarize the incidence of rabies exposure. *P*-value less than or equal to 0.05 at 95% confidence interval was considered as statistically significant.

## Results

### Demographic data

A total of 924 human rabies exposure cases received the anti-rabies post-exposure prophylaxis. Of these, males accounted 55.2% and the median age was 18 year (ranges: 1–80 years). More than half (58.9%, 544/924) of the cases had age range of 1–24 (of which 46.3% were less than 15 years of age) and the remaining were in the age range of 25–54 (32.2%) and > =55 (8.9%). Regarding the residence majority of cases were from rural areas (71.9%, 664/924) (Table 1). Almost all human rabies exposure cases (96.3%) were bitten by domestic dogs and the remaining cases were bitten by cats (14 cases) and donkey (twelve cases) and there were eight cases having contact history with rabies suspected humans. The source of exposure for urban settings was almost 100% domestic dogs.

**Table 1:** Demographic and seasonal distribution of human rabies exposure cases at Addis Alem hospital in 2015 and 2016, Northwest Ethiopia

Age group	Sex			Residence			Season				
	Male N (%)	Female N (%)	Total N (%)	Urban N (%)	Rural N (%)	Total N (%)	Summer N (%)	Winter N (%)	Spring N (%)	Fall N (%)	Total N (%)
< 5	59 (6.4)	63 (6.8)	122 (13.2)	44 (4.8)	78 (8.4)	122 (13)	27 (2.9)	36 (3.9)	37 (4.0)	22 (2.4)	122 (13.2)
5–14	165 (18)	141 (15)	306 (33.1)	86 (9.3)	220 (24)	306 (33)	91 (9.8)	46 (5)	144 (16)	25 (2.7)	360 (39)
15–24	68 (7.4)	48 (5.2)	116 (12.5)	34 (3.7)	82 (8.9)	116 (12)	31 (3.3)	17 (1.8)	48 (5.2)	20 (2.2)	116 (12)
25–34	67 (7.3)	54 (5.8)	121 (13.1)	27 (2.9)	94 (10)	121 (13)	26 (2.8)	34 (3.7)	32 (3.5)	29 (3.1)	121 (13)
35–44	63 (6.8)	25 (2.7)	88 (9.5)	44 (4.8)	44 (4.8)	86 (9.3)	26 (2.8)	15 (1.6)	40 (4.3)	7 (0.7)	88 (9.5)
45–54	42 (4.5)	47 (5.1)	89 (9.6)	141 (15)	75 (8.1)	89 (9.6)	27 (2.9)	18 (1.9)	19 (2.0)	25 (2.7)	89 (9.6)
> = 55	46 (5)	36 (3.9)	82 (8.9)	11 (1.2)	71 (7.7)	82 (8.9)	16 (1.7)	20 (2.2)	40 (4.3)	6 (0.65)	82 (8.9)
Total	510 (55)	414 (45)	924 (100)	260 (28)	664 (72)	924 (100)	244 (26)	186 (20)	360 (39)	134 (14.5)	924 (100)

### Incidence of human rabies exposure

The incidence of human rabies exposure cases during 2015 and 2016 (depicted in Table 2) was 6.5 and 7.5 per 100, 000 population respectively. The overall incidence of human rabies exposure was 7.01 per 100,000 populations (Table 2).

**Table 2:** Incidence of human rabies exposure cases registered and taken anti-rabies post exposure prophylaxis at Addis Alem Hospital by sex (A) and by residence (B) during 2015 and 2016

A				
Year	Sex	Total Population	Number of exposure cases	Incidence per 100,000 population
2015	Male	3,233,545	261	8.1
	Female	3,293,971	162	4.9
	Total	6,527,516	423	6.5
2016	Male	3,289,494	249	7.6
	Female	3,353,380	252	7.5
	Total	6,642,874	501	7.5
B				
Year	Residence	Total Population	Number of exposure cases	Incidence per100,000 population
2015	Urban	1,111,652	117	10.5
	Rural	5,415,864	306	5.6
	Total	6,527,516	423	6.5
2016	Urban	1,172,473	143	12.2
	Rural	5,470,401	358	6.5
	Total	6,642,874	501	7.5

The highest number of exposure cases was reported during spring (360/924, 39%) followed by summer (244/924, 26.4%) (Table 1).

Considering the time of attending the hospital after exposure has been occurred, the majority of human exposure cases came within two weeks of exposure (545/924, 59%) and within one week of exposure (323/924, 35%). There were seven cases (0.8%) and ten cases (1.1%) came within one month and two months of exposure respectively. Most of delayed (came within three weeks and above) exposure cases were from rural settings and there were only four cases from the urban settings who came later.

### Health status of the biting dog

The health status of most dogs (67.3%) involved in biting was unknown (they were stray dogs) and 28.8% were sick: develop the signs of rabid animal within ten days follow up. The remaining dogs were killed immediately (2.4%) and died within one week of biting (1.5%) respectively. The status of donkeys and cats was not known because all cases associated with donkeys and cats were from the rural area and arrived at the hospital late.

## Discussion

Human rabies exposure cases registered for anti-rabies post exposure prophylaxis at Addis Alem government hospital during 2015 and 2016 were reviewed in this retrospective cross-sectional study. A significant proportion of the population (924) in the catchment area of the hospital was found to be exposed to rabies during the study period. The actual number of rabies exposure cases might be more than this figure as there is weak registration and reporting of cases. Even we could not have data regarding the status of the exposed cases after being vaccinated. There was no death reported during the first arrival, but we could not get data after the cases went back their home, there is no strong follow up and tracing system. This shows that human rabies exposure is still among major public health problems in spite of the availability of anti-rabies post exposure prophylaxis that is believed to break transmission besides to save the exposed cases.

More males received the anti-rabies post exposure prophylaxis (52.2%). It reveals that males were more affected than females in the area. This is in agreement with studies in western and northern Ethiopia [15–17], Nigeria [22], Zimbabwe [23] and Tanzania [24]. This might be explained as: males spent most of their time in field works, whereas females are engaged in indoor activities because of religious and cultural influences. Males have also more close contact with dogs than females. In Ethiopia males travel long distance during night times that increase their risk of exposure. But the male to female ratio in this study is nearly 1:1 which is a different result from most studies reported before. This might be because the rural area of the study area in this study is known by agricultural activities and females involved almost equal to the males in these agricultural activities. This might explain that females have nearly equal exposure for biting by rabid dogs as males.

The majority of rabies exposure cases were children less than fifteen years of age. This is in line with previous reports in Gondar [16] and Jimma [17], Ethiopia, Tanzania [24] and World Health Organization report that states children constitute significant proportion of rabies exposure cases from Africa and Asia [25]. This might be because children usually handle and play with domestic animals, including dogs and cat. Besides, nearly all Ethiopian children, especially in rural settings, play in the streets and stay in the field to control sheep, cattle and other animals. These all increase the risk of children being bitten by dogs and other animals. Moreover, youths and adults are aware of the signs of rabid animals and be far away when they face but children can’t protect themselves. However, a study conducted in Tigray [15], Northern Ethiopia showed opposite result where people aged greater than 15 were more affected. The difference might be because of differences in the category of age groups where they categorize in three age groups <=4, 5–14 and > =15.

The incidence of human rabies exposure cases per 100,000 populations was 6.5 and 7.5 in 2015 and 2016 respectively, with the overall prevalence of 7.01 per 100,000 populations. This incidence is higher than a study conducted in Gondar [16]. The presence of areas with better forest coverage in this study area might contribute to the increased incidence. Wild animals like foxes, wolves, hyena, and vampire bats usually live in forest areas and they might have frequent contact with domestic dogs. There is a high possibility to transmit rabies from wild animals to domestic dog during this interaction that again transits to humans. On the other hand, this incidence is much lower than the report in Tigray, Ethiopia [15]. This might be because the study in Tigray was conducted in areas around the Kafta Sheraro national park where different wild animals live that contribute in the transmission of the disease as discussed above.

Rabies is a zoonotic disease transmitted mainly by animal biting. In this study dog bite (96.3%) was the principal source of human rabies exposure. This finding is suported by a previous review [27] and concurrent to studies conducted in Addis Ababa (87.54%) [26], Jimma (90.4%) [17], Gondar (100%) and Tigray (100%) [15], Ethiopia and WHO report and world organization for animal health report [2]. Moreover, 97 and 90% of dog bite human rabies cases using post exposure prophylaxis in Kenya [31] and Zimbabwe [23] respectively have been reported. This is probably because of high human-dog interaction in Ethiopia in general and in the study area in particular. In addition, despite the increased number of dogs in the country, there is no regular and programmed vaccination for dogs. Vaccination is usually administered during outbreaks only. In this study, eight rabies exposure cases were due to human contact/biting. This indicates that there is human to human transmission in Ethiopia. Similar results have been reported in different parts of Ethiopia [17, 21, 30].

In the current study, the majority (71.9%) of exposure cases were from rural areas. There are studies reported similar results [15–17, 28, 29]. This might be related to increased number of stray dogs and the absence of a culture to administer vaccination for dogs and low level of community awareness in the rural areas as compared to the urban areas [3]. In the Ethiopian context, there is at least one dog per household in the rural areas. Dogs are used to protect houses and farmlands from wild animals and unusual human beings. Besides, there is interaction between dogs and wild animals that increase the risk of being infected with *Rabies Virus* and transmit to humans [12, 30]. People in the rural areas also treat exposed individuals by traditional herbal medicines which are not proven for efficacy and effectiveness. This allows exposed individuals to develop the disease and become aggressive to bite other people. On the other hand it indicates exposed people come to health institutions seeking treatment that again shows growing awareness in the population in the study area about the diseases.

High number of rabies exposure cases was recorded during spring (39%) and summer 26.4%). This is in agreement with the report from Tanzania and Nigeria [22, 24]. This is mainly related to the breeding season of dogs. However, studies conducted in Gondar [16] and Jimma [17] report contradictory result where higher numbers of rabies exposure cases were recorded during fall and winter.

There was a statistical difference (*p* < 0.001) regarding the time of arrival in health institutions and residence (Table 3). 8% (52/664) and 1.5% (4/260) of exposure cases from rural and urban areas respectively arrive to the health institution after two weeks of exposure for post exposure treatment. This difference could be due to inaccessibility of treatment sites as there is only one site for around seven million people. Moreover, People in the rural areas usually seek treatment from health institutions when they failed to treat rabies exposure cases with traditional medicines and after the death of one family member and/or neighbor. This may also show awareness difference between the rural and urban community.

**Table 3:** Time of arrival to the Addis Alem hospital for post exposure prophylaxis after human rabies exposure, 2017

Variables		Time of arrival to Addis Alem hospital			Crude OR (95% CI)	*P*-value
		Within two weeks of exposure	After two weeks of exposure	Total		
Residence	Urban	256	4	260	1 5.4 (1.95–15.19)	< 0.001
	Rural	612	52	664		
	Total	868	56	924		
Sex	Male	483	27	510	0.74 (0.43–1.27) 1	0.28
	Female	385	29	414		
	Total	868	56	924		

*OR* = OR of arriving to Addis Alem Hospital after two week of exposure for residence and sex

**Fig. 1:**
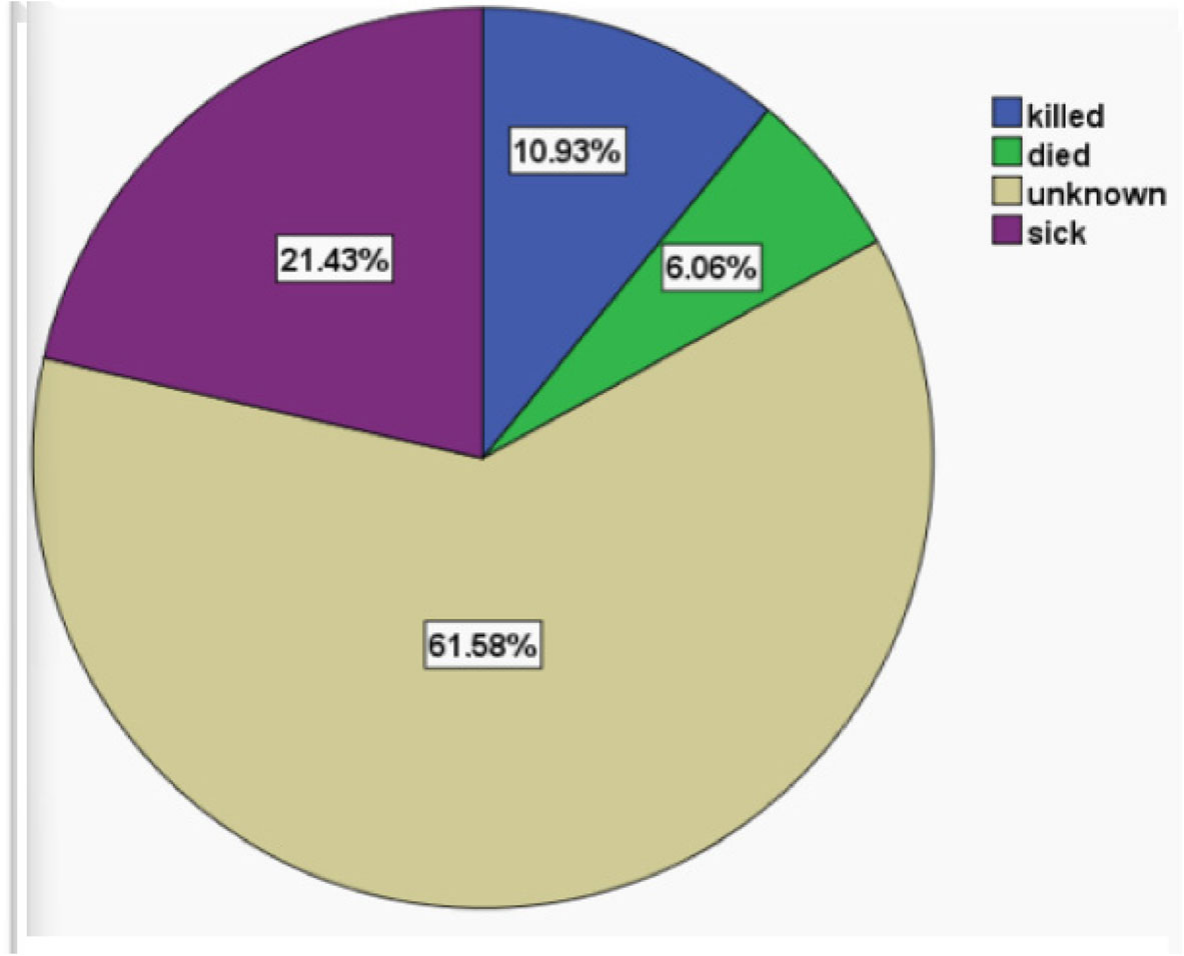
Survival status of rabies suspected dogs during victims visit to Adis Alem hospital between 2016 and 2017, North-West Ethiopia

This study reveals that the survival status of the majority of suspected source animal (dog) is not known (Figure 1). The community usually kills the dogs rather than quarantine and follow up when they show unusual behavior and/or after biting. This hampers to get the actual rabies incidence and further research and intervention strategies. This shows the low level of community awareness and lack of community education as discussed above.

## Conclusion and recommendation

A significant proportion of the population was exposed to rabies virus during the study years in North-west Ethiopia. There was high human rabies exposure rate in children and in the rural community. Dog bite was the principal source of exposure.

This reveals the need to establish organized and scheduled preventive and control strategies in the country at large and in the study area in particular, improve the availability of post-exposure prophylaxis community education and strong and periodic surveillance. Establishing laboratories with the capacity for basic rabies diagnosis and case confirmation is also required. Moreover, we recommend community based follow up studies to understand the status of source animals and the effectiveness of the post exposure prophylaxis.

## Abbreviations

ANRS: Amhara National Regional State
ERCCMHS: Ethics Review Committee of the College of Medicine and Health Sciences
Ml: Milliliter
PEP: Post Exposure Prophylaxis
RABV: Rabies Virus
RNA: Ribonucleic Acid
WHO: World Health Organization
